# Complex Etiology Underlies Risk and Survival in Head and Neck Cancer Human Papillomavirus, Tobacco, and Alcohol: A Case for Multifactor Disease

**DOI:** 10.1155/2012/571862

**Published:** 2012-01-23

**Authors:** Elaine M. Smith, Linda M. Rubenstein, Thomas H. Haugen, Michael Pawlita, Lubomir P. Turek

**Affiliations:** ^1^Department of Epidemiology, College of Public Health, The University of Iowa, Iowa City, IA 52242, USA; ^2^Veterans Affairs Medical Center and the Department of Pathology, Carver College of Medicine, The University of Iowa, Iowa City, IA 52242, USA; ^3^Research Program Infection and Cancer, German Cancer Research Center (DKFZ), 69120 Heidelberg, Germany

## Abstract

Findings are inconsistent about whether tobacco, alcohol, and human papillomavirus (HPV) are two independent HNC risk factor groups that distinguish an infection-associated cancer from a tobacco/alcohol-associated HNC. We found that cancer in the oral cavity risk was greater in HPV-E6/E7 seropositive/heavy tobacco users (adjusted OR = 3.5) than in HPV-seronegative/heavy tobacco users (adjusted OR = 1.4); and HPV-seropositive/heavy alcohol users (adjusted OR = 9.8) had greater risk than HPV-seronegative/heavy alcohol users (adjusted OR = 3.1). In contrast, the risk of oropharyngeal cancer was greater in the HPV-seronegative/heavy tobacco (adjusted OR = 11.0) than in HPV-seropositive/heavy tobacco (adjusted OR = 4.7) users and greater in HPV-seronegative/heavy alcohol users (adjusted OR = 24.3) compared to HPV-seropositive/heavy alcohol users (adjusted OR = 8.5). Disease-specific and recurrence-free adjusted survival were significantly worse in oropharyngeal HPV-seronegative cases with no survival differences by HPV status seen in oral cavity cases. The association between tobacco/alcohol, HPV, and tumor site is complex. There appear to be distinct tumor site differences in the combined exposure risks, suggesting that different molecular pathways are involved.

## 1. Introduction

Human papillomavirus (HPV) is now an established risk factor for head and neck cancers (HNC) [1, 2]. Whether HPV is an independent risk factor of tobacco and alcohol, the other two major causes of tumors at these sites, has not been well clarified [1, 3–5]. It is known that those who are infected with HPV have significantly better survival [6, 7], thus it would be important to clarify the role of these risk factors. Many case-control studies traditionally have employed the anti-VLP-HPV antibody ELISA test to detect a history of HPV infection. Fewer studies have assessed the risk of HNC associated with antibodies against HPV E6/E7 oncoproteins. E6/E7 antibodies are biomarkers of HPV-associated cancer and possibly precancerous lesions and as such should provide a more sensitive measure of HPV-related disease in individuals and in evaluating the association with or independence from tobacco and alcohol. The purpose of this study was to examine HPV E6/E7 antibody status in patients with oral cavity and oropharyngeal HNC, and in healthy controls, for differences in HPV status associated with tobacco and alcohol use. The study also evaluated site-specific HNC survival and recurrence by HPV E6/E7 antibody status compared to HPV tumor tissue status associated with tobacco and alcohol.

## 2. Materials and Methods

### 2.1. Patient Data Collection

Patient characteristics and exclusions have been described previously [1]. Cases with a primary HNC ages 18 and older diagnosed between 2001 and 2004 at the University of Iowa Hospitals, Department of Otolaryngology and the Iowa City Veterans Administration Hospital, were eligible for enrollment. All oral cavity (*N* = 170) and oropharyngeal (*N* = 74) sites were included. There were 244 eligible cases enrolled after exclusions. Gender and 5-year age group frequency matched controls (*n* = 428) were recruited from clinics in Family Medicine and in Internal Medicine at these hospitals who were seeking routine medical care.

Patients signed an institutionally approved informed consent form prior to completing a self-administered risk factor questionnaire that included information about sociodemographics, medical history, tobacco and alcohol use, sexual practices, and history of HPV-related diseases and oral lesions. Prior cancer history, head and neck tumor site, treatments, and staging were collected from medical records and pathology reports. All tobacco and alcoholic products were included and equivalents were used [1].

### 2.2. HPV Laboratory Methods

A blood specimen was drawn from participants to detect HPV at the time of diagnosis prior to cancer treatment for cases or during the interview for controls. Detection of HPV-specific antibodies has been described previously [3–8]. The Luminex procedure detected the presence of HPV E6 and E7 and HPV types were identified with multiplex serology [9, 10]. Multiplex serology uses viral L1 proteins expressed in bacteria as glutathione S-transferase (GST) fusion proteins as antigens [10, 11]. 

Paraffin-embedded tumor tissue was available from 204 cases to evaluate for HPV. Sample preparation, PCR analyses, DNA hybridization, and HPV typing procedures for assessment of tumor tissue were based on a standard protocol [12]. Each PCR reaction included primers to amplify the *β*-globin gene [13] and verified sufficient DNA and adequacy of the PCR amplification. Extracted DNA was PCR-amplified with MY09/MY11 primers [14] to detect HPV and with primer HMB01 to better amplify HPV-51 [15]. Positive samples underwent heminested PCR-amplification with MY09 and GP5+ primers [16]. DNA sequencing was used to determine the HPV types in each specimen, and sequences were compared to GenBank sequences using the BLAST program [17]. High-risk, oncogenic HPV types (HR-HPV) detected in tumor tissue included HPV-16 and 33. 

### 2.3. Statistics

Odds ratios (OR), 95% confidence intervals (CI) and *P* values for risk factors, and pathologic characteristics were adjusted for continuous age, gender, tobacco pack-years, and average drinks per week. Categorical variables included age, education, number of sexual partners, tobacco pack-years, and average number of alcoholic drinks per week. Cut points for moderate and heavy tobacco or alcohol users were based on the overall distributions or median values among HPV-negative cases and controls. ORs and CIs were calculated using multivariate logistic regression analyses. For variables with zero cells, ORs and CIs were generated using logit methods, adjusting for categorical age, tobacco, and alcohol except when limited by small cell sizes. 

Individual and joint effects of tobacco and alcohol stratified by HPV status were examined by tumor site and never users of tobacco or alcohol (reference groups) were compared to moderate or heavy tobacco or alcohol users among cases and controls. Additive joints effects and 95% CIs were assessed using the synergy index [18] while multiplicative interactions were examined by including the appropriate interaction term in the multivariate logistic regression models [19]. Survival curves were generated using Kaplan-Meier methods while Cox proportional hazards models were used to generate hazard ratios (HR) and 95% confidence intervals (CI), adjusted for continuous age and stage of disease (I/II versus III/IV). Nodal involvement, tumor site, and grade were excluded from models due to their high correlation with disease stage. Gender, pack-years, and drinks per week were not associated with survival or recurrence and thus were not included in the models. Analyses comparing HPV E6/E7 assays and tumor HPV DNA included only those patients with results from both assays (*N* = 204 cases). Sensitivity was defined as the percent of HPV DNA positive who were E6/E7 positive; specificity as the percent of HPV DNA negative who were E6/E7 negative; and, concordance as the percent positive for both tests and negative for both tests. Magnitude of agreement was measure by the Kappa statistic. All *P* values were two sided, and values ≤0.05 were considered statistically significant. Statistical analyses were performed using SAS version 9.2. 

## 3. Results 

### 3.1. Risk Factors

The prevalence of HPV-16/18/33 E6 and/or E7 was greater in HNC cases than in controls: 26% versus 7%; it was higher in oropharyngeal cases (64%) compared to oral cavity cases (9%) or controls (7%; Table 1). HPV-16 was the most frequent type detected in cases and controls (25%/5%), followed by HPV-33 (15%/2%), and HPV-18 (0.8/0.9%). Almost all cases with HPV-33 also were detected with HPV-16 (97%) whereas only a third (33%) of controls were. Those detected with HPV-18 were not detected with any other HPV type. The frequency of HPV-16 E6 or E7 only was similar in both cases (20%/18%) and controls (1%/4%). Compared to HPV-seronegative cases, HPV-seropositive cases were significantly more likely to be male. Those with tumors in the oropharynx who were HPV E6/E7 seropositive were more likely to be younger, more educated, and perform oral-genital sex (Table 1). They also showed higher grade and positive nodal status than did tumors in the oral cavity. No differences among controls were identified. 

### 3.2. HPV E6/E7, Tobacco, and Alcohol Status Associated with Site-Specific HNC Risk

We next examined the relationship between tobacco, alcohol, and HPV E6/E7 status by tumor site (Table 2). Compared to controls, those with cancer of the oral cavity had a minimal increased risk among heavy tobacco users whereas the risk of oropharyngeal cancer was increased in both tobacco dose-duration groups and higher in heavy users. Parallel findings were seen for alcohol use although the risks were twice those seen for tobacco exposures, regardless of tumor site. The adjusted risk of tumors in the oral cavity did not increase in the joint heavy tobacco/alcohol users (>30/>21, OR = 5.2) over heavy tobacco users and heavy alcohol users. There was no multiplicative effect associated with tobacco/alcohol among tumors in the oropharynx for heavy users of both, but there was a significant additive effect (OR = 34.6). The independent risk was significantly greater among those with HPV-seropositive status after controlling for tobacco, alcohol, and other risk factors but only in tumors of the oropharynx. 

### 3.3. Site-Specific HNC Risk Stratified by HPV, Tobacco, and Alcohol Status 

Based on the distinct findings by tumor site for tobacco and alcohol risk, data were next stratified by HPV E6/E7 status (Table 3). Compared to controls, cases with oral cavity tumors who were heavy tobacco users had an elevated risk but only among those who were HPV-seropositive (OR = 3.5) but not HPV-seronegative. Those with oropharyngeal cancer had elevated risks at the higher tobacco dose-duration level as well but in both the HPV-seropositive and HPV-seronegative groups, with the odds much higher for the HPV-seronegative group (OR = 11.0 versus 4.7). HPV-seropositive risk was higher in the oropharynx than found in the oral cavity. The risk associated with alcohol use was elevated only in the heaviest users (>21) in oral cavity cases regardless of HPV E6/E7 status, but was higher in the HPV-seropositive group (OR = 9.8 versus 3.1). The ORs for the oropharynx were significantly elevated for both alcohol levels regardless of HPV E6/E7 status although, in contrast to the oral cavity, they were higher in the HPV-seronegative cases. Again there was little difference in risk between the two tumor sites among HPV-seropositive heavy users. 

When both tobacco and alcohol were examined in stratified analyses associated with HPV status, only the heavy tobacco/alcohol (>30/>21) group was elevated for cancer of the oral cavity (Table 3). Consistent with the individual tobacco and alcohol groups, higher risk was particularly seen in those who were HPV E6/E7 seropositive. Again, a different picture was seen for oropharyngeal cancers. Increased risk was shown for almost all tobacco/alcohol groups compared to never users of both. Risks were greater among those who were HPV-seronegative/heavy tobacco/heavy alcohol users than among those who were HPV-seropositive/heavy users of both. CIs were wide due to the expected small rate of HPV E6/E7 positivity in controls.

### 3.4. Comparison between HPV E6/E7 Serology and HPV Tumor DNA 

There were 145 oral cavity and 59 oropharynx cases with both serology and tumor tissue assessed for HPV status. The prevalence of HPV-seropositive in the oral cavity was 10% and 64% in the oropharynx, and 13%/58% for oral cavity/oropharynx tumor HPV DNA-positive status (Table 4). Type-specific concordance between HPV-positive tumors (HPV-16 and/or -33) and HPV-seropositivity was 93% (*K* = 0.9, 0.7–0.99) for the oropharynx and 84% (*K* = 0.2, 0.003–0.4) in the oral cavity. The sensitivity of HPV E6/E7 antibodies to DNA HPV-positive tumors in the oral cavity was 26% and 100% in the oropharynx. 

Moderate smokers (compared to never or to heavy smokers) had elevated ORs for both HPV-seropositive and tumor HPV DNA-positive compared to HPV-negative status. Both moderate and heavy alcohol users had elevated ORs compared to never drinkers for either HPV assessment. For the joint effects of alcohol and tobacco, moderate smokers/drinkers (≤30/≤21) and moderate smokers/heavy drinkers (≤30/≥21) had the greatest odds of being HPV-positive; other risks were similar for serology and tumor tissue. Sample sizes were insufficient to evaluate risk factors for HPV serology and DNA by tumor site.

### 3.5. Survival and Recurrence Associated with HPV E6/E7 and Tumor DNA Status

Table 5 displays the HRs for disease-specific survival (DSS) and recurrence-free survival (RFS) for newly diagnosed patients with survival information (DSS = 144; RFS = 127). Also shown is a comparison of DSS and RFS between HPV tumor and serology among newly diagnosed cases with HPV results available for both outcomes (*N* = 157). Among those who did not survive, 56% died from HNC, 28% from other causes and 16% had an unknown cause. A first recurrence occurred in 30% of all patients. Median follow-up time for DSS was 5.3 years (range: 1 month–10.4 years). Among those who had a DS death, 76% died within 2 years of diagnosis. The median follow-up time for RFS was 5.6 years (range: 2 months–10.4 years). For patients with a first recurrence, 76% occurred within 2 years of diagnosis. Time to recurrence did not significantly vary by HPV E6/E7 status for either site. 

**Figure 1:**
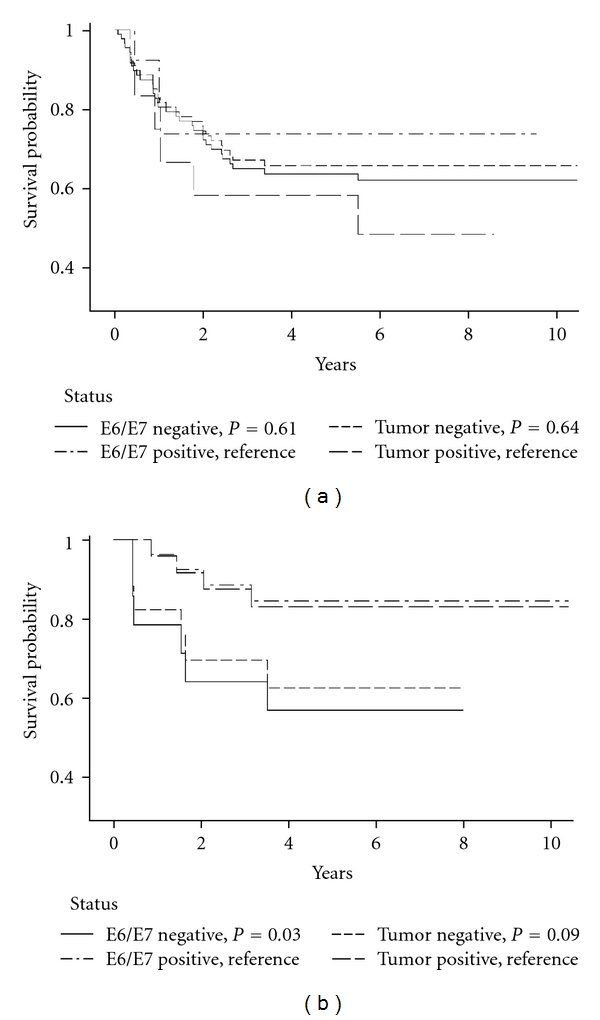
(a) Oral cavity disease-specific Kaplan-Meier survival curves by E6/E7 and tumor DNA status. (b) Oropharynx disease-specific Kaplan-Meier survival curves by E6/E7 and tumor DNA status.

**Figure 2:**
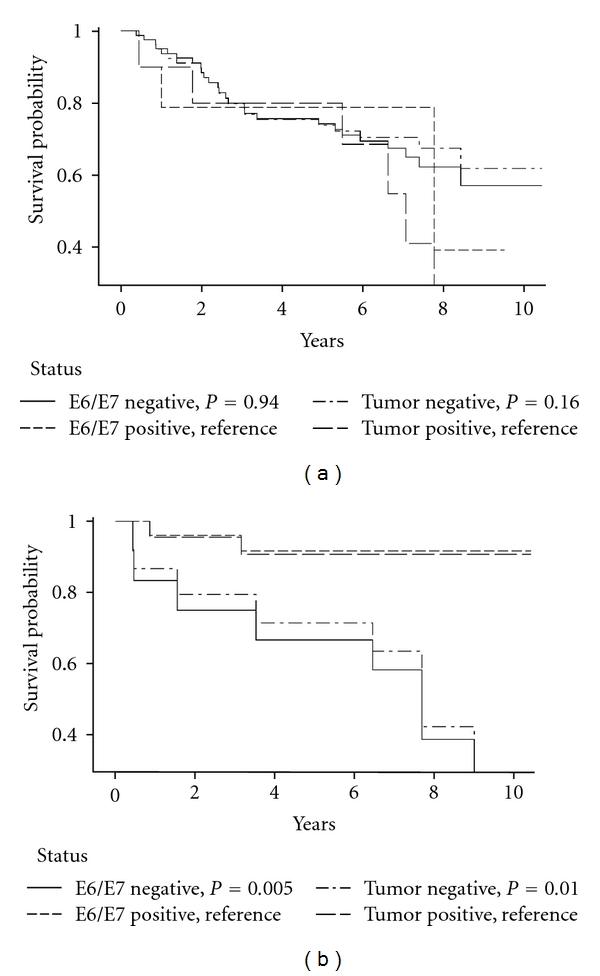
(a) Oral cavity recurrence-free specific Kaplan-Meier survival curves by E6/E7 and tumor DNA status. (b) Oropharynx recurrence-free specific Kaplan-Meier survival curves by E6/E7 and tumor DNA status.

 HPV status was not significantly related to DSS or RFS for oral cavity cancers (Table 5, Figures 1(a) and 2(a)). In contrast, oropharyngeal cancer cases who were HPV-seronegative or DNA-negative reported significantly lower DSS and RFS compared to HPV-positive cases (Table 5, Figures 1(b) and 2(b)). In a DSS model with tumor site, age, stage, alcohol, and tobacco, only age, higher stage, and tumor site were significant predictors of higher mortality risk (oral cavity versus oropharynx: HR = 2.1, 1.03–4.4). 

Concordance between the HPV tests for both sites combined was high for DSS and RFS (88%/88%, *K* = 0.7 both). An examination of sensitivity and specificity for HPV E6/E7 antibodies for HPV DNA status and survival outcomes showed that HPV E6/E7 seronegative status was a strong indicator of DSS and RFS in tumor DNA HPV-negative tumors of the oral cavity (DSS/RFS censored: 90%/94%; death/recurrence: 97%/85%). However, there was weaker sensitivity between HPV-seropositive and HPV DNA-positive outcomes (DSS/RFS censored: 33%/20%; death/recurrence: 33%/67%). In the oropharynx, serology was a much stronger overall indicator and demonstrated strong sensitivity and specificity for tumor tissue status and survival (HPV-negative DSS/RFS censored: 73%/100%; death/recurrence: 100%/100%; HPV-positive DSS/RFS censored: 100%/88%; death/recurrence: 100%/100%; Figures 1 and 2).

## 4. Discussion

The results of this investigation demonstrate that HPV, tobacco, and alcohol represent three independent risk factors for HNC in both the oral cavity and oropharynx. Some previous studies have suggested [3, 4] that HNC would fall into two distinct tumor groups with separate risk factors, HPV or tobacco/alcohol use. Here we show that each risk factor exerted a significant and independent effect by tumor site, although the risks associated with oropharyngeal cancers were consistently higher than that for the oral cavity. This finding is similar to our previous study based on HPV VLP antibody assessment [1], a lifetime indicator of HPV infection. In this study, we evaluated HPV E6/E7 antibodies, a measure of HPV-related current or possibly, precursor cancer lesions. Other investigations have assessed HNC or oropharyngeal tumors and also have shown an independent effect of each of these exposures [5, 20] based on HPV VLP antibodies. 

The current study is one of the few to also examine these risk factors separately for cancers of the oral cavity. In the oral cavity, the risk was more likely to be elevated among HPV-positive/higher tobacco and alcohol level users compared to controls whereas in the oropharynx the opposite was found with HPV-negative/higher tobacco and alcohol level users having a greater risk than the seropositive cases. Although these trends are consistent with our previous investigation based on VLPs, the risks are higher and more evident with the HPV E6/E7 antibodies. Unlike Hafkamp et al. [21] but consistent with Fakhry et al. [22], we found that HPV-positive tumors in never and ever smokers to be unrelated to DSS or RFS (data not shown). Additionally, we found no relationship in HPV-positive or -negative cases and alcohol use. Although the INHANCE analyses did not examine HPV, greater dose and duration of the independent effects of tobacco and alcohol use were associated with higher risk in the oropharynx than in the oral cavity [23]. 

 This also is one of the first studies to examine concordance and sensitivity between E6/E7 serology and tumor DNA for both tumor subsites. We found that they were higher in the oropharynx than in the oral cavity (concordance: 93%/84%; sensitivity: 89%/36%). Previously, Herrero et al. [5] reported better although somewhat lower concordance between HPV E6/E7 serology and tumor HPV DNA in the oropharynx than in the oral cavity: 65% versus 13%. Their corresponding sensitivity was better in the oropharynx than oral cavity as well: 64% versus 14% (*K* = 0.6/*K* = 0.08). The higher percentages in our study associated with the oral cavity may be due to our use of laser microdissection which is more critical for tumors at this subsite. 

 It is unclear why site-specific HPV E6/E7 serology/DNA tumor concordance was consistently high for survival outcomes with the exception of the HPV E6/E7-seropositive/DNA-positive cancers in the oral cavity. Other studies of HPV status in association with HNC survival have focused on oropharyngeal cancer and none has reported on the sensitivity of HPV serology associated with tumor DNA and clinical outcomes [3]. This assessment showed that not only is DS survival better in HPV-positive oropharyngeal cases than in HPV-positive oral cavity cases, but also that they have better RFS than in the oral cavity. Rotnáglová et al. [24] also have shown that DSS is significantly better in HPV E6/E7 seropositive or tumor DNA-positive tonsillar cases followed an average four years, although they did not compare cases with both measures of HPV status. 

 This investigation suggests that while risk of HNC by tumor site is both different between oral cavity and oropharynx, both sites are nonetheless associated with independent effects for each of the three major HNC risk factors. The combined effects alter HNC risk differently in a comparison of HPV-negative versus HPV-positive cases in the oral cavity and the oropharynx. Further, patients with oropharyngeal tumors are different from oral cavity cases not only for the three risk factors but also because they are younger in age, have higher prevalence of heavy tobacco and alcohol use, and are diagnosed at a later stage and with nodal involvement. It is not clear how these differences explain the distinctly different survival and recurrence outcomes found. Additional studies are need that examine HPV in the presence or absence of tobacco and alcohol to evaluate differences in chromosomal alterations and molecular pathways to clarify these risk and survival differences by tumor site.

## Figures and Tables

**Table 1:** Association between HPV^1^ E6 and/or E7 status with head and neck cancer risk factors.

Risk Factors	Oral cavity cases (*N* = 170)			Oropharynx cases (*N* = 74)			Controls (*N* = 428)		
	HPV E6/E7+ * N* (%)	HPV E6/E7− *N* (%)	Adjusted *P* value	HPV E6/E7+ *N* (%)	HPV E6/E7− * N* (%)	Adjusted *P* value	HPV E6/E+ * N* (%)	HPV E6/E7− *N* (%)	Adjusted *P* value
HPV prevalence	16 (9.4)	154 (90.6)		47 (63.5)	27 (36.5)		31 (7.2)	397 (92.8)	

Age^3^	Oral cavity positive versus oropharyngeal positive: *P* value = 0.01; oral cavity negative versus oropharyngeal negative: *P* value = 0.33								
Mean	64.7 (3.6)	61.2 (1.5)	0.35	54.5 (2.1)	58.2 (2.7)	0.28	56.8 (2.7)	58.1 (0.7)	0.63
Range	45–86	21–93		36–82	29–85		18–77	22–93	
≤55	6 (37.5)	55 (35.7)	0.96	29 (61.7)	11 (40.7)	0.05	14 (45.2)	166 (41.8)	0.83
>55	10 (62.5)	99 (64.3)		18 (38.3)	16 (59.3)		17 (54.8)	231 (58.2)	
Gender									
Male	14 (87.5)	79 (51.3)	0.03	40 (85.1)	19 (70.4)	0.04	21 (67.7)	247 (62.2)	0.10
Female	2 (12.5)	75 (48.7)		7 (14.9)	8 (29.6)		10 (32.3)	150 (37.8)	
Education^3^									
<12	5 (31.3)	34 (22.4)	0.58	5 (10.9)	11 (40.7)	0.07	2 (6.5)	52 (13.1)	0.87
12–15	9 (56.3)	87 (57.2)	0.50	32 (69.6)	10 (37.0)	0.51	18 (58.1)	226 (57.1)	0.61
≥16	2 (12.5)	31 (20.4)	Ref	9 (19.6)	6 (22.2)	Ref	11 (35.5)	118 (29.8)	Ref
Tobacco^4^									
Never	3 (18.8)	54 (35.3)	Ref	8 (17.4)	2 (7.4)	Ref	10 (34.5)	150 (37.9)	Ref
>0.0–30	3 (18.8)	41 (26.8)	0.59	19 (41.3)	6 (22.2)	0.85	15 (51.7)	127 (32.1)	0.24
>30	10 (62.5)	58 (37.9)	9.94	19 (41.3)	19 (70.4)	0.37	4 (13.8)	119 (30.1)	0.24
Alcohol^5^									
Never	3 (18.8)	57 (37.3)	Ref	6 (13.0)	2 (7.4)	Ref	14 (45.2)	170 (43.0)	Ref
1–21	5 (31.3)	54 (35.3)	0.92	27 (58.7)	10 (37.0)	0.67	15 (48.4)	166 (42.0)	0.95
>21	8 (50.0)	42 (27.5)	0.64	13 (28.3)	15 (55.6)	0.14	2 (6.5)	59 (14.9)	0.30
Number of Partners									
0-1	6 (42.9)	60 (42.9)	Ref	3 (7.1)	4 (16.7)	Ref	10 (34.5)	122 (32.3)	Ref
2–10	5 (35.7)	59 (42.1)	0.56	23 (54.8)	13 (54.2)	0.55	16 (55.2)	186 (49.2)	0.64
≥11	3 (21.4)	21 (15.0)	0.66	16 (38.1)	7 (29.2)	0.70	3 (10.3)	70 (18.5)	0.21
Oral-genital sex									
Yes	3 (37.5)	39 (49.4)	0.83	24 (92.3)	8 (50.0)	0.02	16 (51.6)	184 (53.8)	0.27
No	5 (62.5)	40 (50.6)		2 (7.7)	8 (50.0)		15 (48.4)	158 (46.2)	
Stage									
I/II	8 (50.0)	58 (39.5)	Ref	3 (6.8)	5 (19.2)	Ref			
III/IV	8 (50.0)	89 (60.5)	0.30	41 (93.2)	21 (80.8)	0.19			
Grade									
Poor/undifferentiated	5 (33.3)	23 (16.2)	0.13	25 (55.6)	3 (12.5)	0.004			
well/moderate	10 (66.7)	119 (83.8)	Ref	20 (44.4)	21 (87.5)	Ref			
Nodal status									
Yes	7 (43.7)	54 (36.0)	0.87	42 (89.4)	20 (74.1)	0.07			
No	9 (56.3)	96 (64.0)	Ref	5 (10.6)	7 (25.9)	Ref			

^1^Positive versus negative for HPV-16 or HPV-18 or HPV-33 E6 and/or E7 status; ^2^adjusted for age, gender, tobacco, and alcohol; ^3^years; ^4^pack-years; ^5^drinks/week.

**Table 2:** Site-specific head and neck cancer associated with tobacco, alcohol, and HPV E6/E7 status.^1^

	Cases		Controls	Oral Cavity versus controls	Oropharynx versus controls
	Oral cavity (*N* = 170)	Oropharynx (*N* = 74)	(*N* = 428)	OR^2^ (95% CI)	OR^2^ (95% CI)
Characteristic	*N* (%)	*N* (%)	*N* (%)		
Tobacco					
Never	57 (33.7)	10 (13.7)	160 (37.7)	1.0	1.0
≤30	44 (26.0)	25 (34.2)	142 (33.4)	0.9 (0.5–1.4)	2.1 (0.9–5.2)
>30	68 (40.2)	38 (52.1)	123 (28.9)	1.5 (0.9–2.4)	5.6 (2.3–13.7)
Alcohol					
Never	60 (35.5)	8 (11.0)	184 (43.2)	1.0	1.0
≤21	59 (34.9)	37 (50.7)	181 (42.5)	1.1 (0.7–1.8)	4.3 (1.7–10.4)
>21	50 (29.6)	28 (38.4)	61 (14.3)	3.6 (2.1–6.2)	11.7 (4.2–32.7)
Tobacco/alcohol					
Never/never	42 (25.0)	3 (4.1)	100 (23.6)	1.0	1.0
≤30/≤21	23 (13.7)	14 (19.2)	72 (17.0)	1.0 (0.5–1.8)	5.2 (1.2–21.7)
≤30/>21	10 (6.0)	8 (11.0)	26 (6.1)	1.4 (0.6–3.5)	15.9 (3.2–80.2)
>30/≤21	22 (13.1)	17 (23.3)	55 (13.0)	1.3 (0.6–2.4)	16.6 (3.9–71.4)
>30/>21	39 (23.2)	19 (26.0)	29 (6.9)	5.2 (2.6–10.5)	34.6 (7.5–158.8)
HPV E6/E7 status^1^					
Positive	16 (9.4)	47 (63.5)	31 (7.2)	1.7 (0.9–3.3)	24.3 (12.9–45.8)
Negative^3^	154 (90.6)	27 (36.5)	397 (92.8)	1.0	1.0

^1^Percentages based on available data; ^2^odds ratio adjusted by age (continuous), gender, HPV status, tobacco, and alcohol; ^3^HPV E6/E7 negative status for all types, E6/E7 positive status for HPV-16, -18, and/or -33.

**Table 3:** Site-specific head and neck cancer stratified by HPV E6/E7, tobacco, and alcohol status.^1^

		Cases		Controls	Oral cavity versus controls	Oropharynx versus controls
		Oral cavity	Oropharynx		OR (95% CI)	OR (95% CI)
HPV E6/E7^2^	Risk factor	*N* = 170 (%)	*N* = 74 (%)	*N* = 428 (%)		
	Tobacco^3^					
Negative	Never	54 (32.0)	2 (2.7)	150 (35.3)	1.0	1.0
	≤30	41 (24.3)	6 (8.2)	127 (29.9)	0.9 (0.6–1.5)	3.2 (0.6–16.6)
	>30	58 (34.3)	19 (26.0)	119 (28.0)	1.4 (0.8–2.3)	11.0 (2.4–51.7)
test for trend *P* value					*0.19*	*<0.0001*
Positive	Never	3 (1.8)	8 (11.0)	10 (2.4)	1.0	1.0
	≤30	3 (1.8)	19 (26.0)	15 (3.5)	0.3 (0.03–2.4)	1.4 (0.4–4.9)
	>30	10 (5.9)	19 (26.0)	4 (0.9)	3.5 (0.5–26.9)	4.7 (1.03–21.7)
test for trend *P* value					*0.008*	*0.01*
	Alcohol^4^					
Negative	Never	57 (33.7)	2 (2.7)	170 (39.9)	1.0	1.0
	≤21	54 (32.0)	10 (13.7)	166 (39.0)	1.1 (0.7–1.8)	5.4 (1.1–25.7)
	>21	42 (24.8)	15 (20.6)	59 (13.9)	3.1 (1.7–5.5)	24.3 (4.9–121.3)
test for trend *P* value					*0.009*	*<0.0001*
Positive	Never	3 (1.8)	6 (8.2)	14 (3.3)	1.0	1.0
	≤21	5 (3.0)	27 (37.0)	15 (3.5)	1.2 (0.2–7.3)	4.1 (1.2–14.7)
	>21	8 (4.7)	13 (17.8)	2 (0.5)	9.8 (0.9–106.9)	8.5 (1.2–60.2)
test for trend *P* value					*0.002*	*0.001*
	Tobacco/alcohol^5^					
Negative	Never/never	40 (23.8)	1 (1.4)	93 (22.0)	1.0	1.0
	≤30/≤21	21 (12.5)	1 (1.4)	63 (14.9)	1.0 (0.5–1.9)	1.9 (0.1–31.1)
	≤30/>21	10 (6.0)	4 (5.5)	24 (5.7)	1.5 (0.6–3.6)	22.8 (2.2-235.2)
	>30/≤21	20 (11.9)	8 (11.0)	53 (12.5)	1.2 (0.6–2.3)	18.7 (2.2–160.8)
	>30/>21	31 (18.4)	11 (15.1)	29 (6.7)	4.0 (2.0–8.3)	56.3 (6.2–512.7)
test for trend *P* value					*0.07*	*<0.0001*
Positive	Never/never	2 (1.2)	2 (2.7)	7 (1.7)	1.0	1.0
	≤30/≤21	2 (1.2)	13 (17.8)	9 (2.1)	0.7 (0.04–12.0)	8.9 (0.96–82.9)^7^
	≤30/>21	0 (0.0)	4 (5.5)	2 (0.5)	0.6 (0.02–17.2)^ 6^	13.7 (0.8–227.9)
	>30/≤21	2 (1.2)	9 (12.3)	2 (0.5)	2.6 (0.1–67.4)	29.3 (2.1–408.7)^7^
	>30/>21	8 (4.8)	8 (11.0)	0 (0.0)	55.0 (0.8–3651.0)^6,7^	18.3 (0.5–627.2)^6,7^
test for trend *P*-value					*0.008*	*0.001*

^1^Percentages based on available data; ^2^HPV E6/E7 seronegative for HPV-16, -18, and/or -33; HPV E6/E7 seropositive for HPV-16, -18, and/or -33; ^3^adjusted for age (continuous), gender, and average drinks/week (continuous); ^4^adjusted for age (continuous), gender, and tobacco pack-years (continuous); ^5^adjusted for age (continuous) and gender, all never groups combined with never/never as reference group; ^6^Logit estimator, 0.5 is added to zero cells; ^7^CMH *P*-value < 0.049.

**Table 4:** Tobacco and alcohol status in head and neck cancer cases by HPV E6/E7^ 1,2^ and tumor HPV status^1^ (*N* = 204).

Risk factor	E6/E7 positive	E6/E7 negative	OR (95% CI)	Tumor positive^3^	Tumor negative^3^	OR (95% CI)
	*N* (%)	*N* (%)		*N* (%)	*N* (%)	
Prevalence	52 (25.5)	152 (74.5)		53 (26.0)	151 (74.0)	
Tumor site						
Oral cavity	14 (26.9)	131 (86.2)	1.0	19 (35.9)	126 (83.4)	1.0
Oropharynx	38 (73.1)	21 (13.8)	17.3 (7.4–40.5)	34 (64.2)	25 (16.6)	8.8 (4.1–19.0)
Tobacco^4^						
Never	8 (15.7)	49 (32.5)	1.0	9 (17.3)	48 (32.0)	1.0
≤30	20 (39.2)	37 (24.5)	2.1 (0.8–5.7)	23 (44.2)	34 (22.7)	3.0 (1.2–7.5)
>30	23 (45.1)	65 (43.0)	1.1 (0.4–3.1)	20 (38.5)	68 (45.3)	1.2 (0.5–3.4)
*≤30 *versus* >30 *			*1.9 (0.8–4.2)*			2.3 (1.1–5.1)
Alcohol^5^						
Never	7 (13.7)	51 (33.6)	1.0	8 (15.4)	50 (33.1)	1.0
≤21	24 (47.1)	56 (36.8)	2.3 (0.8–6.2)	27 (51.9)	53 (35.1)	3.0 (1.2–7.8)
>21	20 (39.2)	45 (29.6)	2.1 (0.7–6.4)	17 (32.7)	48 (31.8)	2.2 (0.7–6.8)
*≤21 *versus* >21 *			*1.1 (0.5–2.5)*	3 (5.8)	10 (6.7)	1.3 (0.6–3.0)
Tobacco/alcohol^6^						
Never//never	4 (7.8)	38 (25.2)	1.0			1.0
≤30/≤21	13 (25.5)	21 (13.9)	3.2 (0.9–12.0)	15 (28.9)	19 (12.7)	4.2 (1.2–14.0)
≤30/>21	4 (7.8)	10 (6.6)	1.8 (0.3–9.3)	5 (9.6)	9 (6.0)	2.6 (0.6–11.8)
>30/≤21	8 (15.7)	24 (15.9)	1.6 (0.4–6.7)	9 (17.3)	23 (15.3)	1.9 (0.5–6.8)
>30/>21	15 (29.4)	34 (22.5)	1.6 (0.4–5.9)	11 (21.2)	38 (25.3)	1.2 (0.3–4.2)

^1^Percentages based on available data; ^2^HPV E6/E7 seronegative for HPV-16, and -33; HPV E6/E7 seropositive for HPV-16 and/or -33; ^3^DNA negative for all HPV DNA types; DNA positive for HPV-16 and/or -33; ^4^adjusted for age (continuous), gender, tumor site (oral cavity, oropharynx), and average drinks/week (continuous); ^5^adjusted for age (continuous), gender, tumor site (oral cavity, oropharynx), and tobacco pack-years (continuous); ^6^adjusted for age (continuous) and gender.

**Table 5:** Adjusted^1^ hazard ratios for disease-specific and recurrence free survival.^2^

Risk Factors	Oral cavity				Oropharynx			
	Disease-specific Survival^1^		Recurrence-free survival^1^		Disease-specific survival^1^		Recurrence-free survival^1^	
	HR (95% CI)		HR (95% CI)		HR (95% CI)		HR (95% CI)	
Patients with HPV serology (*N* = 182)								
		*N* = 116		*N* = 96		*N* = 52		*N* = 43
E6 and/or E7	*n*		*n*		*n*		*n*	
Negative	103 (88.8)	1.3 (0.4–4.2)	86 (89.6)	1.1 (0.3–3.6)	16 (30.8)	5.7 (1.7–20.0)	13 (30.2)	6.7 (1.7–26.1)
Positive	13 (11.2)	1.0	10 (10.4)	1.0	36 (69.2)	1.0	30 (69.8)	1.0
Age^2^		1.02 (1.0–1.1)		1.02 (1.0–1.04)		1.04 (0.96–1.1)		1.03 (0.95–1.1)
Stage								
0/I/II		1.0		1.0		1.0		1.0
III/IV		7.9 (2.8–22.5)		1.5 (0.7–3.1)		1.5 (0.2–11.4)^ 3^		1.3 (0.03–58.8)

Patients with both HPV tumor and serology (*N* = 157)								
		*N* = 100		*N* = 88		*N* = 44		*N* = 39

E6 and/or E7	*n*		*n*		*n*		*n*	
Negative	89 (89.0)	1.4 (0.4–4.5)	80 (90.9)	1.1 (0.3–3.5)	14 (31.8)	4.2 (1.2–15.1)	12 (30.8)	10.4 (2.4–52.4)
Positive	11 (11.0)	1.0	8 (9.1)	1.0	30 (68.2)	1.0	27 (69.2)	1.0
HPV tumor status								
Negative	88 (88.0)	0.8 (0.3–2.0)	78 (88.6)	0.5 (0.2–1.3)	17 (38.6)	3.0 (0.9–10.7)	15 (38.5)	7.3 (1.5–36.4)
High Risk	12 (12.0)	1.0	10 (11.4)	1.0	27 (61.4)	1.0	24 (61.5)	1.0

^1^Adjusted for stage and continuous age; ^2^in years; ^3^odds ratio Logit estimator adjusted for stage and categorical age.
